# Indigenous Peoples: Traditional knowledges, climate change, and health

**DOI:** 10.1371/journal.pgph.0002474

**Published:** 2023-10-13

**Authors:** Nicole Redvers, Paula Aubrey, Yuria Celidwen, Kyle Hill

**Affiliations:** 1 Schulich School of Medicine & Dentistry, Western University, London, Ontario, Canada; 2 Arctic Indigenous Wellness Foundation, Yellowknife, Northwest Territories, Canada; 3 Department of Indigenous Health, University of North Dakota School of Medicine & Health Sciences, Grand Forks, North Dakota, United States of America; 4 Department of Psychology, University of California at Berkeley, Berkeley, California, United States of America; 5 Division of Environmental Health Sciences, School of Public Health, University of Minnesota, Minneapolis, Minnesota, United States of America; McGill University, CANADA

## Abstract

Indigenous Peoples around the globe make up approximately six percent of the global population, yet they sustainably care for around eighty percent of the world’s remaining biodiversity. Despite continued political, economic, and racial marginalization, as well as some of the worst health inequities on the planet, Indigenous Peoples have worked hard to maintain their cultures and languages against all odds. Indigenous Peoples’ close connections to land, water, and ecosystems, however, have placed them at increasing vulnerability from the effects of climate change. With this, the health risks from climate change have unique considerations within Indigenous Nations for both mitigation and adaptation responses that are largely unappreciated. This Indigenous narrative review will synthesis the current climate and health landscape of Indigenous Peoples at a global, high-level scale, including relevant international mechanisms and considerations for Indigenous Peoples’ health. This Indigenous narrative review will also explore and reflect on the strengths of Indigenous traditional knowledges as it pertains to climate change and health.

## Introduction

“*To see from one eye with the strengths of Indigenous ways of knowing*, *and to see from the other eye with the strengths of Western ways of knowing*, *and to use both of these eyes together …*” [1].

One of the most important elements in understanding the climate change and health connection for Indigenous Peoples is to understand their context. It is impossible to understand some of the specific nuances of the climate change and health discourse within Indigenous communities without having a baseline idea of the global realities on the ground and within political arenas. Indigenous Peoples, however, have historically been framed within the literature through a deficit lens, focusing only on the problematization of their issues in addition to the stark health disparities that exist. This deficit narrative has consequently created an image of Indigenous Peoples incongruent with the strengths they provide the global community in this time of great crisis. This deficit context has also most often occurred without a consequent focus on colonization, racism, ever present structural inequalities, and stark political exclusion that has continued to forcibly marginalize Indigenous Peoples around the globe.

For example, despite the small advances in the international arena to address Indigenous demands, needs, and priorities, the COVID-19 pandemic substantially increased the gap of marginalization and political exclusion globally. The pandemic, through structural, political, and other factors, pushed many Indigenous populations into further marginalization through poverty, with a general lack of access to safety, housing, clean water, sanitation, and food security [2–4]. While Indigenous Peoples continue to suffer constant stigmatization, discrimination, lack of political participation, and criminalization in laws, policies, and practices, we also saw many strengths throughout the pandemic, including a resourcing of traditional Indigenous systems for health, care, and recovery throughout the pandemic [5]. These strengths have been less prominent in the international discourse despite there being important lessons learned from Indigenous Peoples regarding their pandemic response [6].

In many cases, climate change has brought deficit-based reporting on Indigenous communities to the forefront (i.e., reporting based on problems and issues alone). Although an understanding of the health risks to communities from climate change, the identification of climate vulnerabilities, and the monitoring of health indicators in response to climate change are incredibly important, it must be balanced with dialogues that uplift Indigenous community strengths and solutions, and that are rooted within Indigenous worldviews. With this, the current review attempts to provide an overview of important dialogues around climate change and health in Indigenous communities from an Indigenous, decolonial, and strengths-based perspective. This topic area is extraordinarily vast, and impossible to address in full in one review. Therefore, we have highlighted some specific items in the current format and have provided ample citations for further topic-specific reading. We focus first on providing a general overview of the global context for Indigenous Peoples, while highlighting important elements around representation, identities, and worldviews. These general features are important to get a foundation of where climate change and health issues are layered on the Indigenous experience, and to better understand general health-related impacts from climate change that is consequently reviewed. We then highlight Indigenous Peoples, climate change and mental health, in addition to elements around food sovereignty as a topic-specific example. Finally, we highlight some case studies throughout to provide on-the-ground examples of the topics covered in the Indigenous review.

### Positionality

As Indigenous scholars we situate ourselves in this Indigenous narrative review with the intent to amplify Indigenous voices and uplift strengths-based dialogues from within Indigenous Nations for the purpose of improving relations between individual, community, and planetary health. Nicole Redvers is an Indigenous scholar and a member of the Deninu K’ue First Nation located in sub-Arctic Canada; Paula Aubrey is an Indigenous health PhD student and an enrolled Tribal member of the Tolowa Dee-ni’ Nation in the United States; Yuria Celidwen is an Indigenous scholar of Indigenous Nahua and Maya descent from the highlands of Chiapas, Mexico; Kyle Hill is also an Indigenous scholar and an enrolled member of the Turtle Mountain Band of Chippewa, and descendent of the Sisseton-Wahpeton and Cheyenne River Sioux Tribes in the United States.

## Indigenous peoples: A global context

To speak about Indigenous Peoples is to discuss plurality, diversity, complexity, and continuous change. Therefore, laying out a general ‎cultural panorama of living dynamic cultures is as complex as its subject. Despite the unique elements of individual Indigenous Nations, this section attempts to give overall considerations on the agreements on Indigenous identities globally, indicating the shared worldviews that have helped establish an international collective voice.

Indigenous demands for justice are a roadmap for how to get past historic discrimination and marginalization in the frameworks of national political and judicial systems [7]. Current established national agreements constrict global movements that have successfully surpassed multi-ethnic differences by the creation of assemblies, consensual processes, and platforms of participation in regional, national, and international political arenas around the world. The emergence of robust international influence has been called “Indigenous diplomacy” [8], which moves through the international community through more horizontal and democratic structures. The Indigenous movement has found consistency within plurality and unity, asserting symbols allowing for the emergence of Indigenous Peoples as political actors in the international community.

Despite the deficiency of available disaggregated data, there are enough data currently available to attempt to draw a more accurate picture of Indigenous Peoples globally. Thus, this section provides indicators to situate the emergence and recognition of the international Indigenous movement for human and collective rights. We have also included a brief account of the principal international frameworks that lay the foundations of the political struggle and demands for Indigenous equity. At the core of these demands are Land ‎rights, addressing the impacts of colonization, and countering the continued colonial narratives that keep Indigenous Peoples as marginalized populations, putting them at sustained and greater risk from climate-related impacts. Therefore, throughout this section there are reflections on the ‎high-level health impacts of the day-to-day challenges Indigenous Peoples continue to face. A short case study of the Maya Indigenous Peoples of southern Mexico and Guatemala additionally illustrates an example of how Indigenous worldviews are embodied, revealing how reverence for Mother Earth is a way to enhance climate-related resilience, social bonds, and the ongoing transmission of cultural values through generations.‎

### Indigenous representation, identities, and worldviews

Indigenous affairs before the 1970s received very little attention in the international community. The International Labor Organization (ILO) had brought forward the 1957 “Convention Concerning the Protection and Integration of Indigenous and Other Tribal and Semi-Tribal Populations in Independent Countries” [9], which was the precedent to the 1989 Convention No. 169 on “Indigenous and Tribal Peoples Convention” [10], but the Indigenous identity as a movement was still in its early development.

The mere subject of “who are Indigenous Peoples and Nations” was still in question at the time. It wasn’t until the United Nations launched a study in 1972 on the problem of discrimination against Indigenous populations [11] that a survey with Indigenous representatives reached a consensus on who and what constitutes “Indigenous.” At the core of these identity pursuits was the inherent and inalienable right of Indigenous Peoples and Nations to self-determination: the vital proclamation that Indigenous Peoples and Nations decide how to be recognized according to their *own* perception and conception, and never according to the values of dominant societies: “No State must take, by legislation, regulations or other means, measures that interfere with the power of Indigenous Nations or groups to determine who are their members” [11]. Thus, for purposes of international action, the working description reached by international consensus is as follows:

Indigenous communities, peoples and nations are those which, having a historical continuity with pre-invasion and pre-colonial societies that developed on their territories, consider themselves distinct from other sectors of the societies now prevailing on those territories, or parts of them. They form at present non-dominant sectors of society and are determined to preserve, develop and transmit to future generations their ancestral territories, and their ethnic identity, as the basis of their continued existence as peoples, in accordance with their own cultural patterns, social institutions and legal system [11].

The central characteristic that guides Indigenous identities derives from a historical and distinctive bond with and belonging to lands and territories. Emphatically, this implies that Indigenous identities are—from its fundamental determination—qualified by the historical colonization, invasion, usurpation, exploitation, and commodification of their lands, territories, resources, air, ice, oceans and waters, mountains, and forests [12]. Being forced into persecution, marginalization, dispossession, and human rights and environmental rights violations such as genocide and ecocide are painful shared characteristics of Indigenous Peoples and Nations globally.

Indigenous worldviews—known as *cosmovisions* in some Nations—guide ways of being through complex systems of traditional knowledges (TK). Traditional knowledge systems (TKS) (see *Glossary*) are based on unique languages, practices, needs, and beliefs transmitted orally through generations. These contextual worldviews are weaved within and throughout Indigenous Lands and Territories and holistically orient all aspects of spirituality; medicine and healing practices; social, economic, and political institutions; governance structures; education systems; ecological knowledges and management systems; and cultural expressions, interactions, and ways of living. The mere capitalization of the words Land and Country has been rightly pointed out to emphasize the Indigenous cultural importance and vital relation with landscapes [13].

At the heart of Indigenous worldviews are the respect for Natural, or First, Law [14], which is based on ethical values of gratitude, reciprocity, responsibility, and belonging [15,16]. Indigenous worldviews have risen as models of diversity integration, promoting positive changes to historic narratives, and altogether new regulatory and development frameworks [17], based on Indigenous values of respect and reciprocity. These values form the basis of many Indigenous Peoples’ identity and the relationship with the natural world [18]. Indigenous worldviews have increasingly been at the forefront of the movement for planetary health and environmental restoration and conservation [19–22].

It is estimated that the Indigenous population globally is more than 476 million people spread across over 90 countries representing more than 5000 cultures [23]. Indigenous Peoples correspond to about 6% of the world’s population and are three times as likely to be in extreme poverty [24]. Along with this striking figure, Indigenous Peoples also have the lowest life expectancy globally, living as much as twenty years fewer than non-Indigenous Peoples [25]. The lack of legally binding frameworks that require States to prioritize global commitments to address Indigenous Peoples’ health, coupled with the climate crisis, intensifies the urgency of Indigenous vulnerable populations (children and youth, women and girls, people with disabilities, migrants, refugees and asylum seekers, 2SLGBTIQ+ peoples, and the elderly), and further hinders the possibility of fulfilling the promise of equality for Indigenous Peoples.

Indigenous Peoples are represented in the seven global geopolitical regions across the world based on how their ecosystems face climate change: Africa, Asia, Central and South America and the Caribbean, the Arctic (and Russian Federation outside Arctic), Central and Eastern Europe and Central Asia/Transcaucasia, North America, and Oceania-Pacific. From the deserts, high mountains ranges and plateaus, the circumpolar circle, rainforest and jungles, coastlines, tundral, and urban areas, Indigenous Peoples are represented. This geopolitical division based on environmental circumstances was a unique introduction brought by the Indigenous movement globally. One great challenge for Indigenous Peoples is that although there is representation in all seven geopolitical regions of the world, this is not always recognized by all state actors, and even in areas where there is state recognition, there can still be many obstacles to full acknowledgment. For example, more than half of Indigenous Peoples are in Asia but failed to be recognized based on unification proclamations in some countries [26], and are even persecuted in others [27]. In fact, Indigenous rights seekers, land and water defenders, and environmental activists are at the highest risk of death threats and death globally [28,29].

Although often not considered in climate and health dialogues, Indigenous languages are an important element of Indigenous health and greater planetary health [30,31]. Indigenous languages are an exquisite repository of Traditional Ecological Knowledges (TEK) (see *Glossary)*, eco-centric cultural and sustainability practices, and Land-based traditions. There are more than 4000 Indigenous languages, which makes up more than half of the world’s approximately 6,700 languages [32]; however, estimates have shown that one Indigenous language is lost every two weeks, with a stark projection of 90% of all languages being lost by 2100 [33]. With this projected loss will go thousands of years of complex ecological knowledge systems [30]. This ongoing threat is a direct consequence of the marginalization and cultural genocide coming from colonization and its colonial practices of assimilation and discriminatory actions.

Drawing attention to this critical language loss, 2022–2032 has been declared the Decade for Indigenous Languages through the Los Pinos Declaration [34]. In it, the international community joins Indigenous Peoples in urging for the integration, preservation, revitalization, and promotion of Indigenous languages, linguistic diversity, and multilingualism-related aspects at the national and international levels. Among the expected actions are access to justice systems, public services, and across public policies in Indigenous languages, ensuring mother tongue–based, bilingual, and multilingual education; access to health care in Indigenous languages; the promotion of Indigenous knowledge technologies and media; and the full support to preserve the intangible heritage expressed through language, including Traditional Ecological Knowledges (TEK).

### Indigenous rights

In keeping with their distinguishing collective identity, Indigenous rights are constituted around the universal demands for autonomy and self-determination; no discrimination; preservation of diversity; and the protection of Lands, Territories, and resources. These make up a corpus of political, land and environmental, legal, economic and socio-cultural, and participatory rights [35]. A number of international frameworks identify these collective rights in international law, the principal of which is the United Nations Declaration of the Rights of Indigenous Peoples (UNDRIP), as well as the aforementioned ILO Convention 169, the American Declaration of the Rights of Indigenous Peoples, the Convention on Biological Diversity, and in many sentences and committees of the Interamerican Commission on Human Rights, the Covenant on Civil and Political Rights, the Covenant on Economic, Social and Cultural Rights, Committee on the Elimination of Racial Discrimination, Committee on the Rights of the Child, and of course the landmark Agenda 2030 on Sustainable Development.

UNDRIP in particular aims to raise Indigenous Peoples in a global context to a political category among the different nations, civil society, and the dominant States. What this means is that UNDRIPs main themes are (i) the right to self-determination, including self-government and legal systems; (ii) the right to be recognized as distinct peoples; (iii) the right to free, prior, and informed consent (FPIC); (iv) the right to be free of discrimination; and (v) the rights to Land and resources (tangible and intangible), including the conservation, protection, use, development, and control under their own laws, traditions, and spiritual relationship [36]. UNDRIP provisions, in addition to the other mechanisms noted (e.g., FPIC), are especially important when considering climate change and health partnerships. There is a strong need to avoid repeating patterns of historic and ongoing knowledge extraction from Indigenous communities without an acknowledgement for their own rights and benefits, including intellectual property rights and Indigenous data sovereignty [37].

It remains a challenge to implement Indigenous rights despite certain States having adopted UNDRIP. Globally, the constitutional level does not effectively scale governance as reflected in public policies, except in three countries in Latin America (Bolivia, Ecuador, and Chile). Variations in judicial situations derive from colonial histories that resulted in public policies of assimilation (from the “mestizaje” in Latin America, boarding/residential schools in North America and Oceania, the governmental homogenization in Africa after the decolonization process of the 1960s, nationalist identities [e.g., Burma, China, France, Russia], to the restriction of Indigenous recognition based on State-specific ideas [e.g., India]) (see *Glossary*). These judicial challenges are great persistent obstacles for Indigenous Nations. In such circumstances, there is a negation of Indigenous presence, depriving the possibility of plurinational governance, as it is perceived as a threat to State identities.

Weathering these opposing forces, Indigenous rights have continued to evolve, gaining visibility and ensuring participation with notable examples from the Arctic, Africa, the Americas, the Pacific, and Asia. Pursuing an integrative, participatory approach, Indigenous Peoples have gained international presence, impacting States and recontextualizing free trade [38]. Indigenous Peoples continue to protest the industrial predatory development against extractivism, and have risen as the frontrunner in environmental management [39] and climate resiliency [40]. The influence of the Indigenous human rights movement in the fight against climate change is multilevel and dynamic, involving cultures, traditional knowledge systems, and strategies for adaptation to defend biodiversity and various diversified systems that make up collective environmental adaptation.

Despite, however, movements forward for Indigenous rights in some areas, there is still a complete lack of equal rights “for the participation of Indigenous Peoples, the right to be heard, the right to make decisions about the status of their lands, territories, and resources, and the right to resolve financial issues directly and without intermediaries”. This includes a lack of direct decision-making power within processes of the United Nations Framework Convention on Climate Change (UNFCC), and with other decisions affecting their lives in relation to sustainable development. For example, the Nature-Based Solutions [41] agenda by 2030 aims to support sustainable development, yet this agenda has so far been deficient in fully implementing the rights of Indigenous Peoples, mainly with FPIC, as well as explicitly addressing how solutions would affect Indigenous lifestyles, lands, and territories. The rights and voices of Indigenous Peoples are also not currently represented inclusively in the Sustainable Development Goals, being mentioned only 6 times in the 169 targets. An Indigenous multicultural approach to sustainability is still missing from its integration in international frameworks, as noted by the Fund for the Development of Indigenous Peoples in Latin America and the Caribbean [42].

**Case study 1.1: Mother Earth and Maya Spirituality**

The Maya of the highlands of Chiapas keep a cosmogonic worldview that explains the generative aspects of life. In practice, this implies carrying a sophisticated calendric system of lunar and solar influences, the first of which guides the seasonal times for ritual and *xukulen* or ceremonial practices. This value system explains all beings in relation to Mother Earth. Therefore, all living beings are represented as a web of relationships in a cosmic living system involving all elements of earth, fire, air, water, and the center that contains them all. Mayan traditional medicine integrates the core of these spiritual conceptions in practices that pursue healing the imbalances that produce disease. Health is a state that is never static. Rather, it is a dynamic fine balance that brings coherence to human participation and action embedded in a cosmic cycle in constant relationship with phenomena. As per many Indigenous Peoples, the Maya perceive well-being as the strength to cultivate connection and nourish interactions with all living beings. These notions of well-being enhance the conservation and restoration of Mother Earth through practice and ensure continuous life ways for all systems involved in planetary health.

## Indigenous health impacts from climate change

Climate change and its impacts on human health disproportionately burden communities experiencing social and health inequities [43]. These impacts are particularly pronounced in those who rely on their immediate environments for subsistence and livelihoods, in addition to those communities experiencing ongoing systems of structural oppression, racism, and legacies of colonialism [44]. Incidentally, the cumulative impact of the social and ecological determinants of Indigenous health and their associated inequities, coupled with historical trauma and loss (see *Glossary*), place Indigenous communities at particular risk for both acute and more chronic and insidious climatic changes [44]. With this context in mind, it is critical to understand the scale of historical trauma and loss, and forced acculturation experienced by Indigenous communities to better situate their risk to health. The risks to health for Indigenous Peoples are therefore especially prominent when considering the anticipated and current impacts of climate change and associated environmental shifts to their land bases.

In the case of American Indian and Alaska Native (AIAN) communities from the United States (U.S.), Farrell and colleagues (2021) estimated the aggregate reduction in cumulative land-base as a result of settler-colonialism and associated federal-Indian policy for Indigenous communities was 98.9%, with 42.1% of tribes losing land claims, altogether [45]. Furthermore, the authors estimated the average forced migration of AIAN communities in the contiguous United States of 239 km, and a maximum forced migration distance of 2,774 km [45]. In all, the authors note that present day AIAN lands experience an increase in calendar days of extreme heat per year (i.e., in excess of 100 degrees F), receive decreased mean annual precipitation, and AIANs occupy lands with less mineral value potential available for economic self-determination. [45]. These findings suggest that not only did Indigenous communities in the U.S. suffer incalculable trauma from their original removal from traditional homelands, but they were also in many cases forcibly relocated to lands exposed to a greater degree of climate change sensitivity and/or risk, as well as lands that would not be as capable of providing economic welfare in the interest of natural resources.

Indigenous Peoples’ ontologies (i.e., ways of being) affirm a dynamic conceptualization of community health, wellness, and collective existence, as intertwined with local ecosystems and planetary health. For this reason, among others noted previously, the threat of climate change is a principal and direct threat to Indigenous communities globally. This solemn truth regarding Indigenous Peoples’ health becomes particularly concerning when we recognize the cumulative impacts and historical trauma and loss associated with legacies of subjugation and violence of Settler-colonialism (see *Glossary)* on Indigenous Nations. From an Indigenous climate-justice perspective, climate change not only represents an existential threat to Indigenous Peoples’ health but is also seen as another layer towards the advancement of White supremist and patriarchal systems over Indigenous ones. Unfortunately, the paradox unfolding represents a scenario where the widely recognized anthropogenic causes of climate change not only target the health of Indigenous Peoples, but also that of White-Settler communities. For this reason, among others, it is still very important to contextualize health risk specifically for Indigenous Peoples while also reinforcing the strengths of Indigenous communities in the interest of environmental justice and climate resilience.

As previously highlighted, climate change exacerbates vulnerabilities to existing social and health inequities, driven by the social and ecological determinants of health. In 2020 alone, 19% of the world’s global land-surface was impacted by extreme drought, which had previously not exceeded 13% [46]. As a result, water security, global food systems and key crops such as rice and maize are impacted, which further affects supply to geographically isolated, and economically destressed communities, further challenging the food insecurity within Indigenous Nations [46]. In addition, Industrialized Nations high on the UN-defined Human Development Index (HDI) (see *Glossary*) account for most pollution-related deaths, as compared to those countries low in industrial activity in the low-HDI group. Even in the most affluent, high-HDI countries, disenfranchised and marginalized populations (including Indigenous populations) bear a disproportionate burden of health impacts due to exposure to air pollution [46]. Similarly, extreme heat exposure disproportionately affects youth, elderly, marginalized and under-resourced communities, particularly those that lack access to adequate health care infrastructure. This is particularly concerning for Indigenous communities when higher temperatures and heatwaves reduce opportunities, frequency, and duration of physical activity, especially beneficial for mental health, while reducing the risk of cardiovascular disease, diabetes, cancer and all-cause mortality [46] Such heat-related risks involve increased health-related hospital admissions and suicidality [46]. As stated earlier, the increasing frequency, duration, and intensity of drought undermine the integrity of the natural environments, which are critical for Indigenous communities and their respective health vis-à-vis interdependence with Land and associated ecosystems. In addition to the devastating consequences of drought, increasing temperatures and extreme heat increase the risk of wildfires in Indigenous communities [45,46]. With respect to overall health impacts, rising air temperatures create favorable conditions for the proliferation of infectious diseases, including food, water, and vector-borne illnesses [46].

At this point in time, the global human health impacts of climate change are undisputable. However, lesser understood are the Indigenous mental health implications, and the dynamic intersection of historical loss and contemporary climate and environmental changes as a direct result of the Anthropocene (see *Glossary*). With this, injuries and mortality related to acute weather events, such as floods and other natural disasters often get attention; however, the associated mental health impacts are often left unattended or misunderstood. When mental health risk associated with climate change is recognized as a function of corresponding social and ecological determinants of health, critical impacts become clear. Etiological pathways through which exposure to acute climate events and associated environmental shifts impact mental health often include acute trauma and general distress [44]. Such impacts may also disrupt the underlying determinants of mental health [47], socio-economic status, physical health, community infrastructure, and social capital [44]. Of particular importance to Indigenous communities is the altered or severed connection to place and corresponding threats to Indigenous knowledge systems, traditional cultural and spiritual practices [44,47]. Therefore, it is not uncommon to lay witness to the complicated grief associated with loss of one’s sense of place due to environmental changes, known as solastalgia (see *Glossary*), or more specific to Indigenous community experiences, ecological grief (see *Glossary*) [44,47,48].

Regarding acuity and chronicity of weather events within Indigenous communities, both acute and short-term weather events, such as storms, temperature, flooding, and seasonality were associated with depression, fear and anxiety, suicide, self-harm, and post-traumatic stress disorder [44]. As mentioned earlier, changes in temperature and precipitation are cited in relation to hospitalizations, suicide, self-harm, substance use, depression, and stress [44]. What is unique to Indigenous communities, however, is the experience of subacute and chronic climatic exposures, such as drought, sea/lake and winter ice extents or changes, changes in wildlife and vegetation, and altered seasons as “cumulative stressors” when experienced alongside historical trauma and loss associated with Settler-colonialism [49]. The cumulative impact of acute and chronic climatic stressors, alone, have been cited as climate-sensitive determinants of mental health (i.e., drivers of food and water insecurity) that could lead to the proliferation of mental-health disorders, increased use of health services, and maladaptive coping mechanisms (e.g., substance use and self-harm) [44]. Additionally, anxiety, sadness, and grief have been recognized as associated with anticipatory environmental changes unfolding in the Anthropocene [44]. Extant literature recognizes the vicarious experience of trauma, sadness, and other emotional experiences for family and friends under duress from climatic events, whether acute or subacute [44]. Finally, despite the experience of historical loss, forced acculturation, and the cumulative impact of a legacy of colonial subjugation, there are deep and ancestral connections to the Land and place that Indigenous communities maintain that are important for a sense of identity, self-worth/concept, and well-being at the individual, familial, and community levels [49,50].

Connection with the land and place grounded in traditional belief systems and knowledges strengthens relationships and feelings of connectedness vis-à-vis cultural and traditional practices [44,47,51]. Whereas environmental shifts leading to ecosystem changes, such as drought, declining sea ice, flooding, etc., disrupt Indigenous communities’ sense of place and have deleterious impacts on cultural and personal identity. These disruptions to cultural and personal identity are linked to substance use, suicidal ideation, general distress, depression, and feelings of disconnectedness from social networks [44,47]. Similarly, climate-induced migration or mobility has been recognized as being particularly distressing in cases where the lived environment no longer supports Indigenous cultures and lifestyles [44,45,47].

Naturally, the implications of anthropogenic climatic changes coupled with industrial disruption to Indigenous community lifeways, a loss of sense of place, and further stress on the social and political determinants of health culminate in many Indigenous communities experiencing a great deal of helplessness and perceive a direct threat to their sovereignty and self-determination. In some cases, hopelessness pervades collective sentiments following land leases to oil and gas, mining, logging, or other projects that undermine environmental health [4,4,4,7,51]. As a result, Indigenous communities often recognize energy infrastructure projects, subsidies to oil companies, and associated extraction as state-sanctioned violence, leaving any chance of climate change adaptation or mitigation opportunities a hopeless endeavor. Thus, enhancing climate resilience and reducing vulnerability to climate change necessitates attention and resources that aim to reduce the burdens of existing social and ecological determinants of health, beginning with regulating the impacts of extractive industries on Indigenous community health at local, national, and global levels [4,9].

**Case study 1.2: Historical loss & health**

As a result of assimilation policies, several Indigenous communities were removed from the Northern Plains and Great Lakes regions of the U.S. and forced to relocate West or “downstream” from the Mississippi and Minnesota Rivers. During this time, land claims were sold to White-Settlers in the territories and homelands historically occupied by these Indigenous communities, and later in the communities they were forced to relocate to. As a result, these Indigenous communities would often be left with topographical elements of a “checkerboard.” Land rights and real estate changed within reservation boundaries, from Indigenous families to that of European Settlers on the same block, or road. To date, inconsistent land and water rights, and checkerboard communities, have severely complicated the ability of Indigenous communities to engage with and practice their Indigenous Traditional Ecological Knowledges (TEK) and associated cultural and spiritual practices. In many cases, such environments, in combination with other social and ecological determinants of health (e.g., unemployment, poverty, lack of adequate housing, lack of access to medical care, nutritional disparities, etc.), were principal causes of rapid acculturation and assimilation of Indigenous communities in the U.S. At the same time, boarding and residential schools run by the federal government and the Christian denominations often forced Indigenous families to surrender their children or face punitive consequences. Such historical events provide the context for the following case example:

Margaret Hummingbird (pseudonym used) is a married 25-year-old female and an enrolled member of an Indigenous community that historically traveled by canoe, with primary subsistence methods that included: hunting, fishing, trapping, and cultivating community gardens. Margaret is the descendent of several boarding/residential school survivors and lives in the city, having relocated from her reservation after graduating from college. She recalls many stories told to her about her namesake, family, and community post-Euro-Settler contact, and throughout the assimilation and termination periods of the U.S. Federal-Indian policies (i.e., 19th–21st centuries). She lives in an apartment with her husband, expecting their first child in the coming months.

Margaret often wonders about the traditional teachings of welcoming a child, how to provide for her family in a sustainable way, and how her people survived during the many periods of colonialism. She continually reflects on the importance of her relationship to the Land. Most importantly, she is proud to reside on the traditional homelands of her Tribal Nations but cannot help but feel the weight of sadness and grief that comes when she prays along the rivers and lakes with the backdrop of manufacturing and commercial industry populating the metro. Although she and her family enjoy the convenience of the city, they are concerned about where they will be able to learn their traditional practices, practice their cultural lifeways, and spirituality. There is a small “Indian Center” in the community; however, building and zoning codes restrict the building of sacred lodges to pray at the Indian Center. Critically, she feels a profound sense of urgency and a poor self-concept, engaging in negative self-talk for not knowing her Indigenous language, and not being able to teach this to her children.

She also frequently corresponds with her mother, Estelle. Since childhood, Estelle has lived on the reservation that her tribe was forcibly relocated to. Estelle often recalls the transition to commodity foods, or federal subsistence boxes received during the time when her people moved away from hunting and gathering, fishing, and gardening. She, too, feels a sense of grief for the loss experienced in her community, especially when Margaret asks her for traditional teachings or language teachings to help support her family. Estelle remembers growing up going to Catholic mass, attending first communion, etc. She does not remember how to make a cradleboard, how to plant a garden, or how to say “I love you” in her traditional language. She also remembers the toxic waste that the paper mill would allow to run-off into the river, and how the water never “tasted quite right” after that. Sadly, she also remembers that there was once Manoomin, or wild rice, in the lakes and they would harvest this food on the water when she was young. She remembers picking berries in the summer—this is her favorite memory.

## Climate change & indigenous food systems mitigation strategies

As we have highlighted previously, Indigenous Peoples globally face extreme poverty, food insecurity, and greater rates of adverse health outcomes when compared to the general population [25]. Indigenous Peoples even from high income countries such as Canada, Australia, Aotearoa (New Zealand), and the United States have a life expectancy that is 5–10 years fewer than other citizens [52]. Human health outcomes are intrinsically linked to the health of the ecosystems, but also the agricultural systems in which they are positioned [53–55]. Nutrition is a key determinant of health for all people; however, it is one of the least focused areas of the determinants of health [56]. As has been highlighted, climate change impacts the health of Indigenous Peoples physically, mentally, and spiritually; however, physical health impacts also include diet-related diseases as a result of decreased access to traditional foods [57].

Indigenous Peoples’ traditional food systems prior to colonization, protected Indigenous Peoples health and their lifeways [58]. These food systems were built upon generations of Traditional Ecological Knowledge (TEK) of the lands, water, seasonality, and sustainable agricultural practices [59]. Diets and cultural life-ways depend upon a variety of native fungi, plant and animal species for consumption, medicinal properties, and ceremonial practices [60]. Traditional food systems are therefore highly dependent upon a healthy environment and ecosystem, as they rely heavily on biodiverse sources of plants and animals [61].

There is also a deep interconnectedness between environmental health, traditional food consumption, and cultural practices within Indigenous communities. Indigenous Peoples have a unique relationship with places, ecological process, and all forms of life both living and non-living, and therefore access to traditional foods are directly tied to cultural, physical, emotional, psychological, and spiritual health as noted [60]. Reciprocity is a term used to describe these relationships with traditional foods, and is characterized by respect and responsibility for wildlife, the land, and natural resources [60]. Indigenous Peoples have a responsibility to tend to the land in which they are centered, not only for the health of the land itself but for the health of the community. When a portion of the environment is unhealthy, for instance water contamination, this will affect all aspects of health for both the land and the people. The consumption of traditional foods, although important for subsistence, is vital to cultural connectedness that occurs through storytelling, ceremony, harvesting, and sharing of resources within community [60].

Strategies to overcome the adverse effects of climate change on food systems include increased promotion of Indigenous food sovereignty (see *Glossary*), listening to Indigenous voices when dealing with mitigation and adaptation strategies around food systems, and utilization of TEK in the restoration of ecosystems. Practicing Indigenous food sovereignty allows Indigenous communities to increase access to traditional and healthy foods while also reducing dependence upon unhealthy processed foods [61]. Food sovereignty therefore supports many public-health initiatives, in addition to supporting resilience against climate change, including addressing diet-related health disparities by focusing on food system changes that are culturally acceptable and address Indigenous health concerns that are self-identified [62].

Indigenous health and nutrition are also affected by the ability of Indigenous Peoples to access resources. Access to resources is greatly influenced by the relationships with legal authorities including national governments [60]. Policy restrictions around traditional food access, for example, affects Indigenous health by limiting the ability of Indigenous People to practice their culture and traditional ways, and therefore build resilience against climate change [60]. Restrictive access to traditional foods through policies are further layered on restricted access through land-rights constraints and land degradation due to climate change and related processes. Here we amplify further two contexts that are important when considering Indigenous health in the context of climate change.

### Food insecurity & climate change

Food insecurity is defined as the lack of stable access to nutritious food, and is a global public-health priority due to its close relationship to mental and physical health [62]. The lack of quality food contributes to malnutrition and poor health outcomes [63]. Food insecurity is associated with several diet-related chronic diseases, including obesity, diabetes, and hypertension [25]. Indigenous Peoples face disproportionately high rates of food insecurity as a byproduct of settler-colonial activities, including forced relocation and degradation of traditional subsistence patterns [62]. As forced relocation includes Indigenous Peoples being stripped from their homelands, the result is complete disconnection from the Land and its food sources. Food insecurity is a global health problem that affects the entire planet, with particular focus on countries with agricultural systems that are sensitive to climate change [63]. Climate change results in variability of rainfall, temperature, drought, and floods [63]. These effects have amplified negative impact on agricultural systems, food systems, and Indigenous ways of being, further increasing levels of health disparities.

### Ecosystem health

The ability of Indigenous Peoples to practice their culture and traditional ways of being is dependent upon the health of the Land as noted previously. As ecosystems and water resources continue to be exploited and converted for other uses, the availability of a variety of sources of traditional foods are put at increasing risk within communities [60]. Varying shifts in harvesting as a result of climate change, and the misalignment between the presence of necessary pollinators can also impact traditional food production and access [60]. Further impacts on the productivity of remaining ecosystems include disease, pollution, invasive species, and adverse management actions [60]. We further emphasize that Indigenous traditional food systems and cultural ways are closely tied to the health of the land, and therefore Indigenous health indicators will be vulnerable to sustained changes in local climate patterns.

### Mitigation & adaptation

“We are trying to get back to an intact world. Climate change can be a vehicle for that because of the awareness it brings to so many about limitations in the current management practice.”

-Ron Reed, Cultural Biologist and Traditional Dipnet Fisherman, Karuk Tribe

Climate and health adaptation is characterized by the implementation of key strategies aimed at protecting health from the effects from “climate-related hazards” and differs from climate change mitigation [64]. Mitigation efforts focus on the reduction of greenhouse gas emissions. Climate change adaptation plans are being used by Indigenous Peoples and tribes to address the climate-related health threats [64]. In the development of adaptation plans, a “values-driven” community-informed approach allows Indigenous People to define health, establish health priorities, and determine necessary actions to improve health [65].

Food sovereignty is a promising approach for improving health [25] in the context of climate change. This approach focuses on revitalization of local food systems, resulting in an increase of access to healthy and culturally appropriate foods [62]. There are seven key indicators for supporting community capacity building to support food sovereignty and traditional food consumption including: (1) access to resources, (2) production, (3) trade, (4) food consumption, (5) policy, (6) community involvement, and (7) culture [62]. Current global food systems, are neither sustainable nor healthy and have detrimental effects on the land, water, soil, air, and other natural resources used to support them [61,66]. Sustainable food systems are those whose aim is to limit the negative effects on the environment while providing adequate nutrition and food security that improves socio-economic welfare [66].

Inclusion of Indigenous voices in climate adaptation and mitigation strategies are vital [67–69] as climate change clearly affects Indigenous food security, lifeways, and health [70–72]. Four key strategies determined to be critical for climate change adaptation are: (1) strengthening Indigenous governance autonomy and authority, (2) promoting knowledge sharing for adaptation practices within and among communities, (3) promoting adaptive co-management among governance scales, and (4) developing learning platforms for climate impacts and adaptive strategies [73].

Traditional Ecological Knowledge (TEK) has become a prominent player in the advancement of climate science globally. TEK can provide additional information about the natural world that is not readily available through western science ways [74–7,6] TEK systems have been established primarily through oral teachings across generations, and is becoming a powerful source of information capable of ensuring continued use of traditional foods and sustainability generally [60]. TEK, while primarily utilized within Indigenous communities, may help to inform strategies for adaptation to climate change that are useful for all people [60]. TEK will continue to play an important role within diverse research and practice approaches only when Indigenous rights are acknowledged and protected [13]. By ensuring Indigenous-led processes are enabled and supported, collaboration between Indigenous Peoples and non-Indigenous entities in a culturally appropriate manner will be better ensured [60].

**Case study 1.3: Climate Adaptation in Action—Karuk Tribe of Northern California, United States**

The Karuk Department of Natural Resources Strategic Plan [57] states:

“Since time immemorial, the Karuk have lived in the Klamath-Siskiyou Mountains in the mid-Klamath River region of northern California. With an Aboriginal Territory that includes an estimated 1.38 million acres, the ancestral people of the Karuk resided in more than one hundred villages along the Klamath and Salmon Rivers and tributaries. Thriving with a subsistence economy supported by rich natural endowments and a strong culture-based commitment to land stewardship, Karuk environmental management has shaped the region’s ecological conditions for millennia. Through carefully observing natural processes, the Karuk have developed traditional management regimens based on a landscape-level ecosystem approach. Self-described as ‘fix the world people,’ the Karuk continue ceremonies that restore balance and renew the world” (Table 1).

**Table 1:** Karuk Climate Adaptation Plan [57].

WHO?	Karuk Tribe
WHAT?	Karuk Climate Adaptation Plan which calls for more traditional fire practices, acknowledging the effects of climate change and need for utilization of TEK for the protection of the local community and culture.
WHERE?	Karuk Tribes ancestral territory spans 1.38 million acres of Northern California, an area that experiences catastrophic wildfires that are driven by poor land management, fire suppression, and global warming.
WHY?	This showcased adaptation plan calls for more prescribed fire and cultural burns. Traditional Karuk fire practices have been criminalized for the last 150 years. The adaptation plan seeks to re-establish prescribed fire necessary during the appropriate timeframes throughout the year as was done for thousands of years prior to colonization. In 2020, Happy Camp California experienced a catastrophic fire that burned many homes and traditional gathering areas. Therefore, the need for prescribed fire is an urgent and ongoing necessity. There are excessive fuels on the land within Karuk Tribes’ ancestral territories that have not been consistently managed as they span across various land-ownership types.
WHEN?	This adaptation plan was created in March 2019
HOW?	Immediate adaptation needs: • Increased coordination with other departments • Development of emergency management programming • Smoke monitoring • Expansion of individual and community cooling centers & air purifying resources Long-term adaptation: • Planning for increased occurrences of physical and mental health conditions resulting from climate change Additional adaptations: • Expansion of Karuk sovereignty and management authority • Revitalization of cultural indicators for fire applications • Increased public education and outreach • Increased outreach and coordination with agency and NGO partners • Development of Air Quality and Climate Adaptation Programs Restoration of Fire Regimens • Potential to inform both climate adaptation and mitigation efforts • Reduction in high severity fires and potential for reduction in forest emissions

## Conclusion

“*In the vision for a healthy future*, *it is important to create the conditions that enable the overcoming of the dissonance between ‘being in nature’ (i*.*e*., *nature that surrounds us) and ‘being of nature’ (i*.*e*., *nature that embodies us)*” [77].

Despite continued political, economic, and racial marginalization, as well as some of the worst health inequities on the planet, Indigenous Peoples have worked hard to maintain their cultures and languages against all odds. Indigenous Peoples’ close connections to land, water, and ecosystems, however, have placed them at increasing vulnerability from the effects of climate change. In this narrative review we provided an overview of important dialogues around climate change and health relevant to Indigenous communities. As we have noted previously, this topic area is extraordinarily vast, and impossible to address in full in one review. Therefore, we have chosen to highlight only some specific items we feel are best addressed in the current format and have provided ample citations for further topic-specific reading for those interested in increasing their learning.

The health risks from climate change have unique considerations within Indigenous Nations for both mitigation and adaptation responses. Additionally, there are unique elements and strengths gleaned from Indigenous traditional knowledges as it pertains to climate change and health; however, only with a clear recognition of Indigenous Peoples sovereignty and rights in all geopolitical areas. There must be continued and sustained dialogues and movements that uplift Indigenous community strengths and solutions as we work towards a healthy, inclusive, and just planet.

## Glossary

***Anthropocene Epoch*:** “… is an unofficial unit of geologic time, used to describe the most recent period in Earth’s history when human activity started to have a significant impact on the planet’s climate and ecosystems” [78].

***Assimilation*:** A series of efforts to assimilate Indigenous Peoples into mainstream Western or dominant societies.

***Ecological Grief*:** The grief felt “… in relation to experienced or anticipated ecological losses, including the loss of species, ecosystems and meaningful landscapes due to acute or chronic environmental change” [79].

***Historical Trauma and loss*:** is “… the collective emotional and psychological injury over the life span and across generations, resulting from a cataclysmic history of genocide” [80].

***Human Development Index (HDI)*:** is a composite index that captures the substantive contributions to population-level health according to three indices or dimensions: life expectancy, education, and per capita gross national income [81].

***Indigenous Food sovereignty*:** “Indigenous food sovereignty is a specific policy approach to addressing the underlying issues impacting Indigenous …” Peoples and their ability to respond to their own needs for “… healthy, culturally adapted Indigenous foods. Community mobilization and the maintenance of multi-millennial cultural harvesting strategies and practices provide a basis for forming and influencing ‘policy driven by practice’” [82].

***Indigenous data sovereignty*:** is “… in its proclamation … the right of Indigenous Peoples to govern the collection, ownership, and application of data, [and] recognises data as a cultural and economic asset” [83].

***Settler-colonialism*:** “… is a distinct type of colonialism that functions through the replacement of Indigenous populations with an invasive settler society that, over time, develops a distinctive identity and sovereignty” [84].

***Solastalgia*:** “… is the distress that is produced by environmental change impacting on people while they are directly connected to their home environment” [85].

***Traditional ecological knowledges (TEK)*:** “… is the on-going accumulation of knowledge, practice and belief about relationships between living beings in a specific ecosystem that is acquired by Indigenous Peoples over hundreds or thousands of years through direct contact with the environment, handed down through generations, and used for life-sustaining ways” [86]. “TEK is inherently multidisciplinary in that it links the human and the nonhuman, and is the basis not only for Indigenous concepts of nature, but also for concepts of indigenous politics and ethics” [87]. TEK plays a role as a collaborative concept, inviting people to engage in a process of respectful learning about significant differences, as TEK is not a homogenized knowledge system, but vastly contextual to place and people [88].

***Traditional knowledge systems (TKS*):** Are systems of knowledge, know-how, skills and practices that are developed, sustained, and passed on from generation to generation within a community, often forming part of its cultural or spiritual identity [89].
